# Identifying barriers and facilitators to implementing advance care planning in prisons: a rapid literature review

**DOI:** 10.1186/s40352-020-00123-5

**Published:** 2020-09-21

**Authors:** Ashley Macleod, Divya Nair, Ekin Ilbahar, Marcus Sellars, Linda Nolte

**Affiliations:** Advance Care Planning Australia, Austin Health, PO Box 5555, Heidelberg, Victoria 3084 Australia

**Keywords:** Advance care planning, Advance directives, Prisoners, Corrections, End-of-life care

## Abstract

**Background:**

Limited information is available describing advance care planning (ACP) within correctional facilities, despite its increasing relevance due to the ageing population in prisons and the high rates of complex medical comorbidities. In Western countries, self-determination with respect to making future medical decisions is a human right that prisoners do not lose when they are remanded into custody. ACP enables individuals to plan for their health and personal care so their values, beliefs and preferences are made known to inform future decision-making, for a time when they can no longer communicate their decisions. This paper examines the limited academic literature relating to ACP within prisons to identify barriers and facilitators that influence the uptake of ACP and advance care directive (ACD) documentation. Common themes related to ACP in a correctional setting were extracted and synthesised to produce a high-level analysis of barriers and facilitators influencing ACP uptake for prisoners within a correctional setting.

**Results:**

Six articles met the selection criteria and reported on the experience of ACP and ACDs in prisons; five from the United States of America and one from Switzerland. Three dominant themes were identified, with related subthemes: system-level factors, attitudes and perceptions, and ACP knowledge and comprehension. Barriers to ACP and ACD implementation were more prominent in articles than facilitators.

**Conclusions:**

Limited academic literature regarding the implementation and experience of ACP in prisons is available. The dominance of barriers identified in studies highlights key challenges for improving ACP uptake in correctional settings. Further research is required to understand the barriers, enablers, and attitudes to ACP in prisons.

## Background

Advance care planning (ACP), palliative care, and end-of-life care is increasingly relevant within correctional facilities due to the ageing population in prisons and the high rates of complex medical comorbidities in older prison populations (Australian Institute of Health and Welfare, 2019; Enggist, Møller, Galea, & Udesen, 2014). Self-determination with respect to making future medical decisions is a human right that prisoners do not lose when they are remanded into custody, and is the fundamental principle guiding ACP. However, limited academic literature examines the experience of ACP for prisoners and correctional health care staff.

The process of ACP is described as planning for one’s future health and care where the person’s values, beliefs and preferences are made known to inform future decision-making if the person can no longer communicate their decisions (Working Group of the Clinical Technical and Ethical Principal Committee of the Australian Health Ministers’ Advisory Council, 2011). The goal of ACP is to align the care a person receives with their documented medical treatment preferences for care (Buck et al., 2019). Noted benefits of engaging in ACP include: reduced burden on acute hospital services, greater use of hospice at end-of-life, medical care that focuses on patient comfort rather than prolonging life at any cost, better alignment of treatment received with a patient wishes, greater patient satisfaction with care, and reduced anxiety and stress for surviving relatives .

In Australia, jurisdictional governments have committed to ACP and improved end-of-life care via legislation, policy, and service reform. All states and territories have legislative instruments allowing competent individuals to legally appoint a substitute decision-maker for if and when they lose decision-making capacity (Haining, Nolte & Detering, 2019). Ideally, ACP conversations should result in the person’s values or medical treatment preferences being documented in an advance care directive (ACD) (Buck et al., 2019). The signed ACD should then be shared with the treating team, substitute decision-maker(s) and any others involved in the person’s health care, and will only take effect when the person loses full decision-making capacity. These documents are legally binding and are a right of all individuals including those remanded into custody, and health professionals have legal obligations to access and enact a person’s ACD where one exists and is relevant (Fountain, Nolte, & Wills, 2018).

Currently, little is known about the experience of prisoner autonomy in relation to medical decision making in Australia, the uptake of ACDs, or the broader experience of ACP in correctional settings. ACD prevalence rates in the general Australian population currently sit between 14 and 30% (Detering et al., 2019; White et al., 2014), suggesting ACP documentation rates among Australian prisoners is also likely to be low. It is important to understand what factors encourage or prevent the uptake of ACP in correctional facilities.

To our knowledge, no study has summarised the available academic literature relating to ACP and prisoners to identify the factors influencing the uptake of ACP and ACD documentation in correctional settings. This information is critical to understand how ACP can be effectively implemented within a correctional setting. This rapid literature review therefore aims to examine the factors influencing the uptake of ACP and ACD documentation in prisons.

## Methods

In early 2020, a rapid review of relevant academic literature published in the last 10 years was conducted to identify the barriers and enablers influencing the uptake of ACP or ACDs in correctional facilities. Data from relevant articles were extracted and synthesised to develop descriptive themes of barriers and enablers from the perspectives of prisoners and correctional healthcare providers. The rapid review was informed by the Preferred Reporting Items for Systematic Reviews and Meta-Analyses (PRISMA) approach to systematic reviews (Liberati et al., 2009; Moher, Liberati, Tetzlaff, & Altman, 2009), Enhancing Transparency of Reporting the Synthesis of Qualitative Research (ENTREQ) framework (Tong, Flemming, McInnes, Oliver, & Craig, 2012), and the work of Thomas and Harden (2008). This approach is also supported by a similar scoping review protocol (Hand, Mitchell, & DeGregory, 2016).

### Data sources and search strategy

Three researchers (DN, EI, AM) searched for articles published between 2009 and 2019 in PubMed, Medline and Embase databases. Terms used in the search included ‘advance care planning’, ‘advance directives’, ‘prisons*‘and other related terms. Papers were included if they were qualitative or quantitative empirical research studies published in English addressing ACP and ACD practices or interventions in prisons, investigated barriers associated with implementing ACP programs in prisons, or were studies investigating prisoners and health care providers’ experiences with ACP or ACDs. Papers were excluded if they discussed palliative care, end-of-life care, terminal illness, or hospice care for the prisoners without the inclusion of ACP or ACDs.

**Fig. 1:**
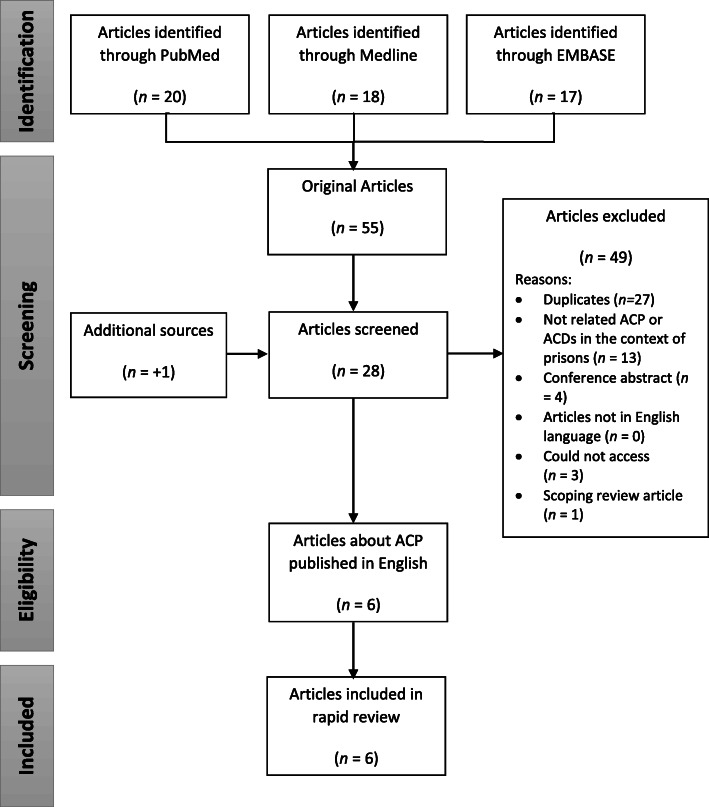
PRISMA diagram describing rapid review search results

Initial database searches were conducted across PubMed, Medline and Embase. After removing duplicates, abstracts were screened by two researchers (DN, EI) using the inclusion criteria, and potentially relevant articles were flagged for full text review (Fig. 1). Reference lists of all articles included in the full text review were screened by title, and any potentially relevant articles found were added to the full text review. Where needed, input from the research team was used to determine the relevance of any articles screened using the full text that the two researchers were unsure whether to include.

### Data extraction and analysis

Data were extracted from each included article and recorded in a single Excel file. These data included: authors, year of publication, the country where the study was conducted, research setting, sample demographics including participant type (staff/prisoner), sample size, age range and sex of participants, and the research approach and measures used in the study (Table 1). Thematic analysis was used to identify common themes describing barriers or facilitators related to ACP implementation in a correctional setting. Thematic analysis involved reading and re-reading manuscripts to identify and extract key messages. Extracted data were examined collectively to identify commonalities in results across manuscripts. Common themes were organised into a framework describing the identified barriers and facilitators to ACP in prison settings. Any issues during the data extraction and thematic analysis process were raised with at least two other authors for further discussion. Themes were then synthesised to produce an overall picture of the barriers and facilitators to ACP in a correctional setting in the existing literature.

**Table 1:** Profile of included articles

Authors	Year	Country	Setting	Sample	Size (***n***)	Age Range	Sex	Approach	Measures
Handtke V, Wangmo T.	2014	Switzerland	12 Swiss prisons	Prisoners	35	51–71 years (mean = 61 years)	30 M, 5F	Individual semi-structured interviews	Prisoners were asked about end of life, death, and dying, demographic and incarceration information, general physical health information, presence of diseases, mental health status and symptoms, medications, substance use, visits to medical services, and problems with activities of daily living. Interviews were followed by a geriatric evaluation consisting of five standardised tests. Interview guide used by researchers was developed using existing literature and expert opinion and pilot-tested with two older adults from the community and further adapted after the first four interviews with older prisoners based on their feedback.
Sanders S, Stensland M, Dohrmann J, Robinson E, Juraco K.	2014	USA	State medical classification center for 3 Midwestern male prisons	Correctional healthcare staff	3	n.a	n.a	Observation study as part of an intervention program	Staff-level data were identified through detailed observational (field) notes by researchers during the implementation process of the study
				Prisoners who were cognitively intact, older, frail, or reasonably thought to die within the next 12 months.	20	25–79	20 M, 0F		Prisoner-level data were identified during facilitated ACP discussions between trained prison staff and prisoners using a detailed data collection tool with 11 primary areas of focus: prisoner views on life support/life-sustaining procedures, end-of-life wishes, health literacy, decision-making and decision-makers, most meaningful aspects of life, questions raised by prisoners, emotions expressed, concerns related to ACP, significant issues raised, nonverbal communications between the ACP facilitator and prisoner, and non-verbal cues made by the ACP facilitator.
Sanders S, Stensland M.	2018	USA	As per Sanders, Stensland, Dohrmann, Robinson, & Juraco, 2014 (above)	Prisoners as per Sanders et al., 2014 (above)	20	25–79	20 M, 0F	As per Sanders et al., 2014 (above)	As per Sanders et al., 2014 (above)
Sanders S, Stensland M, Juraco K.	2018	USA	As per Sanders et al., 2014 (above)	As per Sanders et al., 2014 (above)	20	25–79	20 M, 0F	As per Sanders et al., 2014 (above)	As per Sanders et al., 2014 (above)
Stensland M, Sanders S.	2016	USA	As per Sanders et al., 2014 (above)	Prisoner composite characters	3	40–84	3 M (composites)	Case study	Three composite offender descriptions were developed using data collected during a larger study (see Sanders et al., 2014) to conduct a critical analysis and discussion of ethical issues related to ACP and end-of-life expereinces in prisons
Ekaireb R, Ahalt C, Sudore R, Metzger L, Williams B.	2018	USA	Four prisons in 2 states and 1 large city jail in a third state.	Correctional healthcare providers	24	n.a.	8 M, 16F	Individual, semistructured telephone interviews	Open- and closed-ended questions related to prisoner’s comfort discussing ACP, timing and process for ACP conversations, barriers encountered at patient-, provider-, and system-levels, whether the correctional setting influenced ACP conversations, and what interventions would help facilitate ACP.

## Results

Initial database searches generated 55 articles from PubMed, Medline and Embase. After removing duplicates, abstract screening using the inclusion criteria identified articles for full text review (Fig. 1). One additional source was identified during screening of reference lists and was added to the articles for full text review. In total, 29 articles were screened for their inclusion in the review using the full text. After examining the full text against the inclusion and exclusion criteria, six articles were included in the review.

All six studies used qualitative methods to explore ACP in correctional settings. Five studies were conducted in the United States (Ekaireb, Ahalt, Sudore, Metzger, & Williams, 2018; Sanders et al., 2014; Sanders & Stensland, 2018; Sanders, Stensland, & Juraco, 2018; Stensland & Sanders, 2016), and one was conducted in Switzerland (Handtke & Wangmo, 2014). The correctional facilities sampled in the US included four state prisons (Ekaireb et al., 2018; Sanders & Stensland, 2018), one jail (Ekaireb et al., 2018) and one state medical classification centre (Sanders et al., 2014; Sanders et al., 2018). All facilities sampled in the US included only male facilities. Correctional facilities sampled in Switzerland included 12 prisons and included both male and female facilities (Handtke & Wangmo, 2014).

Four papers included participants who were prisoners (Handtke & Wangmo, 2014; Sanders et al., 2014; Sanders et al., 2018; Sanders & Stensland, 2018), and one paper included composite characters that had been developed through semi-structured interviews with prisoners (Stensland & Sanders, 2016). Three papers included participants who were healthcare professionals, including nurses (Ekaireb et al., 2018; Sanders et al., 2014; Sanders et al., 2018), social workers (Ekaireb et al., 2018; Sanders et al., 2014; Sanders et al., 2018), and physicians (Ekaireb et al., 2018). No papers included participants who were non-healthcare correctional staff.

**Fig. 2:**
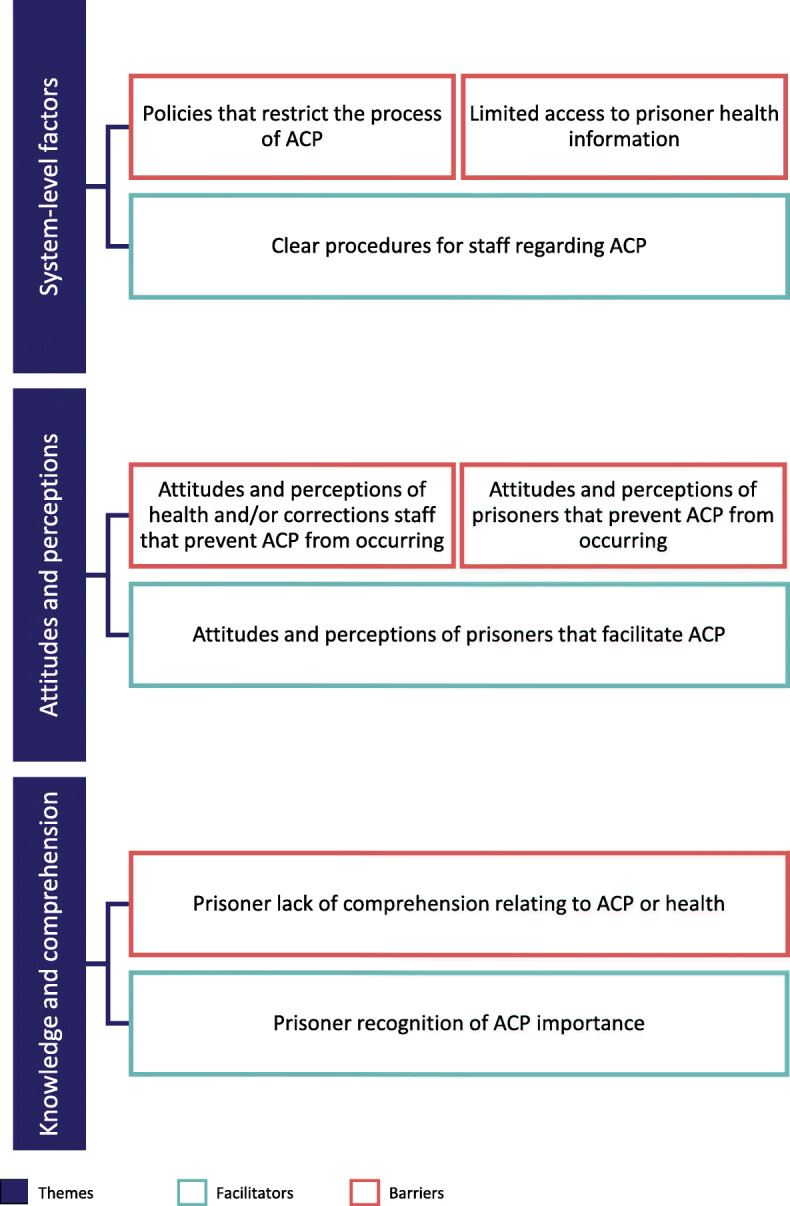
Major themes and related subthemes describing barriers and facilitators to ACP in a correctional setting

Papers focused primarily on prisoner and healthcare worker experiences and attitudes towards ACP and end-of-life experiences in prisons. Three themes containing between two and five sub-themes were identified reflecting barriers and facilitators that impact ACP implementation within a correctional setting. These themes included: system-level factors, attitudes and perceptions, and ACP knowledge and comprehension (see Fig. 2).

### System-level factors

Five of the six included studies described ways the corrections system can impact the process of ACP and the ability for healthcare staff to enact the preferences of prisoners (Ekaireb et al., 2018; Handtke & Wangmo, 2014; Sanders et al., 2014; Sanders et al., 2018; Stensland & Sanders, 2016). Barriers related primarily to how restrictive prison policies impacted ACP processes (Ekaireb et al., 2018; Handtke & Wangmo, 2014; Sanders et al., 2014; Sanders et al., 2018; Stensland & Sanders, 2016) and issues related to inadequate access to prisoner health information in medical records (Ekaireb et al., 2018; Sanders et al., 2014; Sanders et al., 2018; Stensland & Sanders, 2016). Facilitators included access to medical facilities that allow prisoner ACDs to be executed (Sanders et al., 2018) and the presence of well-developed ACP policies and procedures (Sanders et al., 2014). System-level barriers are discussed first, before describing system-level facilitators for ACP in prisons.

#### Policies restricting the process of ACP

Restrictive prison policies impacted the ACP process by restricting how and when ACP discussions could occur (Ekaireb et al., 2018; Handtke & Wangmo, 2014; Stensland & Sanders, 2016), limiting the types of documents and preferences considered acceptable (Ekaireb et al., 2018; Stensland & Sanders, 2016), and by restricting the ability for healthcare workers to action a prisoners’ ACD (Ekaireb et al., 2018; Sanders et al., 2014). Health practitioners indicated restrictive policies that isolated prisoners made ACP discussions difficult. These difficulties were primarily attributed to prisoners becoming distracted by concerns about how to engage their family in the ACP process, or because policies required health care providers to conduct these conversations while separated from prisoners by a physical barrier (Ekaireb et al., 2018). Prisoners blamed strict prison policies for preventing them from identifying a substitute decision-maker, from appointing another inmate as their substitute decision-maker (Stensland & Sanders, 2016), and for preventing dying inmates from being able to remain connected with their family (Handtke & Wangmo, 2014). In one paper, restrictive prison policies prevented prisoners from completing ACP documentation unless they have a terminal illness, or unless the document was generated outside of the prison facility (Ekaireb et al., 2018). Prison policies also limited the ability of health professionals to honour prisoners’ end-of-life preferences (Sanders et al., 2014) and undermined the type of trust needed by prisoners to know their preferences will be respected (Ekaireb et al., 2018). For example, health practitioners referred to laws and policies preventing them from being able to respect the wishes of inmates who did not want to die in prison (Ekaireb et al., 2018).

#### Limited access to information in prisoner health records

Both health practitioners and inmates discussed the restricted flow of health information between health practitioners and prisoners. Prisoners felt unable to move forward with ACP because they had limited access to information about their health status or treatment options (Sanders et al., 2018). Health practitioners also indicated ACP discussions with prisoners were hindered by procedural restrictions limiting the type of information they could share with prisoners, particularly when these details may pose a security risk (Stensland & Sanders, 2016). Health practitioners also reported that ACP was neglected in prisons because of a lack of standardised documentation (Ekaireb et al., 2018) and a lack of document storage processes providing easy access to (or transfer of) prisoners’ medical orders or ACP preferences between systems and/or other facilities (Ekaireb et al., 2018; Sanders et al., 2014).

#### Medical infrastructure limitations preventing ACDs from being enacted

Restrictions limiting the ability of health practitioners to comply with prisoner preferences were also identified in terms of whether the required medical facility infrastructure was available. For example, Stensland and Sanders (2016) reported a prisoner’s end-of-life request to be transferred to a former prison facility so they could die surrounded by friends was not granted because the requested facility was not equipped to deal with the medical needs of the prisoner (Stensland & Sanders, 2016).

#### Clear procedures for staff regarding ACP

Health practitioners felt ACP could be facilitated in prisons if well-formed processes allowing a prisoner’s medical records to move with them between facilities were developed and correctly followed (Ekaireb et al., 2018). Similarly, proactive education of relevant staff (including prison security and external facility healthcare workers) about the risks of not following medical document transfer processes facilitated the ability of medical professionals to provide appropriate care to prisoners (Sanders et al., 2014). ACP was considered more effective in prisons where processes allowing a prisoner’s medical records to move with them between facilities were well-formed and correctly followed by staff (Sanders et al., 2014). One paper also suggested staff provide clear and consistent instruction to prisoners about what types of preferences can or cannot be carried out under current Department of Corrections policy at the start of the ACP process to limit the inclusion of unachievable goals in prisoner ACDs (Sanders et al., 2014).

### Attitudes and perceptions

Five papers described the different impacts the attitudes and broader perceptions of health practitioners, corrections staff and prisoners can have on the process of ACP (Ekaireb et al., 2018; Handtke & Wangmo, 2014; Sanders et al., 2014; Sanders & Stensland, 2018; Stensland & Sanders, 2016). All health practitioner attitudes and perceptions functioned as barriers to the ACP process, or in ways that prevent treatment in line with a prisoner’s preferences (Ekaireb et al., 2018; Sanders & Stensland, 2018; Sanders et al., 2014, p. 328). Only prisoners identified any attitudes or perceptions that facilitate the ACP process in a correctional setting (Sanders et al., 2018).

#### Attitudes and perceptions of health and/or corrections staff preventing ACP from occurring

The attitudes and perceptions expressed by healthcare workers and other corrections staff in the included articles functioned as barriers to ACP in a correctional setting. Some health practitioners resisted engaging in ACP discussions because they were worried about triggering fears of dying in prison for the inmate (Ekaireb et al., 2018). Some staff were also resistant to engaging in ACP processes because this would mean an increased workload and learning new processes (Ekaireb et al., 2018; Sanders & Stensland, 2018; Sanders et al., 2014, p. 328), or reported that ACP was not considered a priority in their facility (Ekaireb et al., 2018). One article also indicated some corrections staff and correctional healthcare workers did not consider ACP to be relevant to prisoners, or felt prisoners were not entitled to ACP (Ekaireb et al., 2018). Some corrections staff saw the role of a prison physician was to keep prisoners alive to ensure they serve their full prison term; these individuals also appeared to feel that engaging in ACP would allow prisoners to avoid serving their full term by opting out of treatments for early death (Ekaireb et al., 2018). Health practitioners also noted a lack of trust by prisoners towards healthcare staff and other correctional staff was a barrier to ACP activities (Ekaireb et al., 2018). This lack of trust presumed by health practitioners was attributed to prisoner fears that ACP conversations were evidence the state was attempting to speed up the dying process (Ekaireb et al., 2018).

#### Attitudes and perceptions of prisoners preventing ACP from occurring

Prisoner attitudes highlighted a lack of trust in the prison health care system (Ekaireb et al., 2018; Handtke & Wangmo, 2014; Sanders et al., 2018; Sanders & Stensland, 2018; Stensland & Sanders, 2016). This lack of trust included a perceived lack of concern by health practitioners and corrections staff (Handtke & Wangmo, 2014; Sanders & Stensland, 2018), and fears their preferences would not be respected (Ekaireb et al., 2018; Handtke & Wangmo, 2014; Sanders et al., 2018; Sanders & Stensland, 2018; Stensland & Sanders, 2016). Prisoners also felt a focus on following procedure and a lack of concern by health practitioners and corrections staff meant their healthcare would be compromised and their wishes ignored (Ekaireb et al., 2018; Handtke & Wangmo, 2014; Sanders et al., 2018; Sanders & Stensland, 2018; Stensland & Sanders, 2016). ACP was also negatively impacted by the difficulty prisoners had in disclosing they were dying to others because they feared corrections staff and fellow inmates would view them as vulnerable and may take advantage of their ‘weakened condition’ (Sanders & Stensland, 2018).

#### Attitudes and perceptions of prisoners that facilitate ACP

Although no health practitioner or correctional staff attitudes and perceptions were identified that facilitate the ACP process, Sanders et al. (2018) reported some prisoners felt relieved and lucky to have an ACP or have taken part in ACP, and engaging in ACP gave them a greater sense of agency and control over the dying process (Sanders et al., 2018). Having an ACD or physician order for life-sustaining treatment (POLST) also gave prisoners a sense of relief their preferences were known (Sanders et al., 2018).

### ACP knowledge and comprehension

Four articles referenced aspects of knowledge and comprehension related to ACP processes and/or prisoner health (Ekaireb et al., 2018; Handtke & Wangmo, 2014; Sanders et al., 2018; Stensland & Sanders, 2016). Health practitioners focused predominantly on describing issues related to a lack of health literacy or mental health issues in prisoners (Ekaireb et al., 2018; Stensland & Sanders, 2016), while prisoners described their understanding of the importance of ACP in a correction setting (Ekaireb et al., 2018; Handtke & Wangmo, 2014; Sanders et al., 2018; Stensland & Sanders, 2016).

#### Prisoner lack of comprehension relating to ACP or health

Health practitioners indicated low health literacy in prisoners was a barrier to ACP, particularly when prisoners did not understand their diagnosis, state of illness, prognosis, and treatment options (Ekaireb et al., 2018; Stensland & Sanders, 2016). Mental health issues (such as schizophrenia or personality disorders) were also discussed by health practitioners as barriers preventing prisoners from being able to make informed health decisions and participate in the ACP process (Ekaireb et al., 2018; Stensland & Sanders, 2016). This lack of prisoner comprehension was described as a key contributor to the difficulties experienced by health practitioners engaging prisoners in ACP conversations (Ekaireb et al., 2018; Stensland & Sanders, 2016).

#### Prisoner recognition of ACP importance

Although prisoners did not mention their ability to comprehend their medical needs and prognosis, prisoners still appeared to understand the importance of ACP in documenting their preferences (Handtke & Wangmo, 2014), and were grateful and relieved to have engaged in ACP (Sanders et al., 2018). Prisoners also demonstrated awareness that if they are not able to communicate their preferences and did not have a substitute decision-maker recorded, the state medical director or warden would act as their substitute decision-maker (Sanders et al., 2018).

## Discussion

This rapid literature review investigated the barriers and facilitators to implementing ACP and ACDs in prisons, showing limited published research in this area to date. Six studies originating in the US and Switzerland identified factors influencing the uptake of ACP for prisoners, correctional staff, and service providers across three primary themes. System-level factors included limits posed by restrictive prison policies, difficulty accessing prisoner health information, and the quality of policy, processes and training related to ACP within the correctional facility. Attitudes and perspectives included negative attitudes and perceptions of healthcare professionals and correctional staff, a lack of trust by prisoners towards the corrections system and staff, and positive attitudes towards ACP by prisoners who had engaged in ACP processes. Factors related to ACP knowledge and comprehension reflected recognition by prisoners of the importance and value of ACP in correctional settings.

Health practitioners and prisoners felt policies restricting the ACP process and the ability for healthcare workers to enact a prisoners’ ACD were a prominent barrier to ACP (Ekaireb et al., 2018; Sanders et al., 2014; Stensland & Sanders, 2016). Although limiting prisoner ACP preferences that conflict with laws or pose a security risk may be necessary, policies limiting ACP to prior to incarceration ignore the UN Standard Minimum Rules for the Treatment of Prisoners (the Nelson Mandela Rules; United Nations, 1990). These rules protect the human rights of prisoners to health care equivalent to that in the community. Given the limited contact many older prisoners have previously had with the healthcare system (Enggist et al., 2014), it is unlikely that many prisoners would know about ACP or have had the opportunity to develop an ACD. Yet the high incidence of comorbidities in the geriatric prison population highlight the importance of ensuring prisoners are able to engage in ACP and actively engage in discussions about their medical care.

Difficulties accessing prisoner health information differed between prisoners and health practitioners. Health practitioners were concerned prisoners would have difficulty understanding their diagnosis or treatment plan because of low health literacy or mental health issues (Ekaireb et al., 2018; Stensland & Sanders, 2016). Unfortunately, it is unclear from this review whether ACP is more likely to occur where health practitioners perceive the prisoner as capable of understanding their prognosis, or whether healthcare practitioners avoid engaging prisoners in ACP conversations because they anticipate comprehension issues in prisoners.

Health practitioners were also concerned that having ACP discussions with prisoners would trigger fears of dying in prison (Ekaireb et al., 2018). Health practitioner concern regarding patient reactions to ACP conversations are also widespread in a community setting (Boddy, Chenoweth, McLennan, & Daly, 2013; De Vleminck et al., 2013), and health practitioners report that a patients’ fear of mortality often prevents them initiating ACP discussions in hospitals (Boddy et al., 2013). In contrast, prisoners felt health practitioners did not provide them with enough information about their health or treatment options to participate in ACP effectively (Stensland & Sanders, 2016).

Both prisoners and health practitioners described prisoner lack of trust in correctional health practitioners and/or the corrections system as barriers to engaging in ACP in prisons. Having limited knowledge of treatment options and disease progression, low levels of education and poor health literacy also prevent ACP outside of correctional environments (Boddy et al., 2013; Lovell & Yates, 2014; Nouri et al., 2019), and people with lower levels of education and poor health literacy are more likely to distrust health practitioners and the health system (Boddy et al., 2013; Nouri et al., 2019). These common barriers to ACP in community and correctional settings suggest trust is a universally important part of ACP processes, and that combating poor understanding of health and ACP should involve providing information in easy-to-understand materials (Nouri et al., 2019).

Prisoners reported being worried their preferences would not be respected (Ekaireb et al., 2018; Handtke & Wangmo, 2014; Sanders et al., 2014; Sanders et al., 2018; Sanders & Stensland, 2018; Stensland & Sanders, 2016), and feared being targeted by others because of perceived weakness (Sanders & Stensland, 2018). Similar fear-based resistance to ACP is also present in the community. For example, health practitioners have reported some patients resist engaging in ACP because they worry having an ACD would make it easier for others to take control of their future healthcare decisions (Boddy et al., 2013).

In contrast, prisoners who had engaged in ACP processes reported having an ACP or POLST gave them a greater sense of agency and control over the dying process, and a sense of relief that their preferences were known (Sanders et al., 2018). In community settings, engaging in ACP can provide patients with peace of mind about their future healthcare (Boddy et al., 2013; Brinkman-Stoppelenburg, Rietjens, & Van der Heide, 2014). As such, providing education about substituted judgement and the roles and responsibilities of a substitute decision-maker is a vital part of promoting the uptake of ACP in both community and correctional settings.

Health practitioners were primarily focused on issues related to producing and accessing ACP documentation in prisons (Ekaireb et al., 2018; Sanders et al., 2014). Similar problems are also present in community healthcare settings (Boddy et al., 2013; Hagen et al., 2015; Lund, Richardson, & May, 2015). Strategies proposed to address these problems in a community healthcare setting may also be relevant within a correctional setting. Previously proposed strategies include increasing population awareness of ACP, having organisational leaders emphasise the high priority of ACP for staff, providing staff with training about ACP including simple scripts to use to promote comfort during conversations, and using an electronic records system to track and store ACP documentation (Hagen et al., 2015).

Concerningly, some corrections staff did not recognise prisoners right to ACP, and were resistant to engaging in ACP to avoid additional workloads (Ekaireb et al., 2018; Sanders et al., 2014; Sanders & Stensland, 2018). However, prisons have a responsibility to prisoners to provide healthcare that is at least the equivalent of the health care available in the general community (United Nations, 1990) and the right to self-determination concerning future medical treatment is not lost when a person is remanded into custody (Enggist et al., 2014; Hand et al., 2016; Johnstone & Kanitsaki, 2009). As such, it is vital that prisons adequately educate their staff on the rights of prisoners to obtaining equivalent health care, including access to ACP.

Despite the limited research investigating the experience and uptake of ACP in correctional settings, this review has several implications for the corrections system. In particular, the results argue for changes to policy to ensure prisoners have access to information resources, are able to have conversations about and document their preferences for care, and for the resultant documents to be stored in their health record so that healthcare staff can access and enact these documents at point of care. There is also evidence that ACP uptake in prisons could be improved by including ACP performance markers in regular reporting exercises.

A recently published report released by the Australian government identified similar barriers to ACP in Australian prisons as those identified in this report (Australian Healthcare Associates, 2020). Likewise, many of the identified themes are present in research related to ACP uptake in the wider community. This overlap between community and correctional healthcare settings suggests approaches developed to improve ACP uptake in the general population may be useful in correctional settings. However, additional research is needed to determine what components of existing interventions designed to increase uptake of ACP in the community are transferrable to a correctional setting while working within the bounds of the law and correctional policies. Research is also needed to examine the prevalence and quality of ACDs in prisons, investigate whether the experience of ACP for prisoners in Australian prisons align with the experiences identified in this review, and determine whether the end-of-life experiences of prisoners align with their stated preferences.

The research identified in this review included the perspectives of corrections healthcare professional. However, it was unclear whether these health professionals also provided healthcare outside of prisons. As such, future research could examine whether differences exist in the perspectives of health professionals working in prisons exclusively and those working across both community and correctional settings. Research examining the perspectives of correctional officers regarding ACP for prisoners would also be beneficial, as the perspectives of correctional officers in this review were described by participating health professionals and not directly sourced from correctional officers.

Interventions to improve ACP uptake in prisons are likely to require different approaches for staff and prisoners. For staff, improving ACP uptake in prisons may require proactive education and training about the importance and relevance of ACPs for prisoners and improving organisational leadership and processes within prisons to emphasise the importance of ACP within a correctional setting (Hagen et al., 2015; Sanders et al., 2014). For prisoners, it may be beneficial to help them develop their communication skills so they can more effectively engage in ACP discussions and communicate their preferences to others. Including behavioural change interventions such as the behaviour change wheel (Michie, Van Stralen, & West, 2011) in interventions would also provide additional opportunities to customise the intervention to reflect the specific barriers and facilitators present within the target facility.

### Limitations

Searches were limited to three databases to ensure a rapid turnaround for the review. Including a wider range of databases may have identified additional articles to include in this review. The rapid review focused on identifying and synthesising the academic literature relating to ACP in prisons, rather than answering specific research questions. However, the results and discussion are limited by the small number of articles available, with five of the six studies based in the US. This dearth of literature makes it difficult to identify universal themes describing the experience of ACP in prisons.

## Conclusion

Limited academic literature related to the implementation and experience of ACP in prisons is available, and primarily reflects the US experience. Barriers and facilitators related to the implementation of ACP in prisons were grouped into systems-based factors, attitudes and perspectives of staff and prisoners, and understanding and knowledge of ACP by staff and prisoners. ACP and substitute decision-making appears poorly integrated into correctional health currently and barriers exist at the system, staff, and prisoner levels. As the number of older prisoners dying from natural causes is increasing, improving ACP uptake in prisons is essential to ensure prisoner medical treatment preferences are respected. Further research is needed to better understand the attitudes, perspectives and experience with ACP for prisoners, prison-based health practitioners, correctional officers, and health practitioners providing care to prisoners. While there are limited studies, the participation of prisoners in such studies demonstrates their willingness to engage in research and issues regarding ACP, ethical treatment, and end-of-life care.

## Data Availability

Data sharing is not applicable to this article as no datasets were generated or analysed during the current study.
